# Acquired perforating dermatosis associated with risankizumab

**DOI:** 10.1016/j.jdcr.2024.01.023

**Published:** 2024-02-02

**Authors:** Munira Alhadlg, Ghadah Alhammad, Abdulaziz Madani

**Affiliations:** Department of Dermatology, College of Medicine, King Saud University, Riyadh, Saudi Arabia

**Keywords:** acquired perforating dermatosis, interleukin-23 inhibitor, risankizumab

## Introduction

Perforating dermatoses encompass a category of papulonodular disorders characterized by the penetration or removal of dermal connective tissue through the epidermis.^1^ Acquired perforating dermatosis (APD) is typically identified in the context of an underlying systemic condition, such as diabetes mellitus and chronic renal failure.^2^ In this case report, we describe the occurrence of APD in connection with the administration of risankizumab, an anti-interleukin (IL)-23 agent, in a 45-year-old man diagnosed with generalized pustular psoriasis.

## Case report

**Fig 1:**
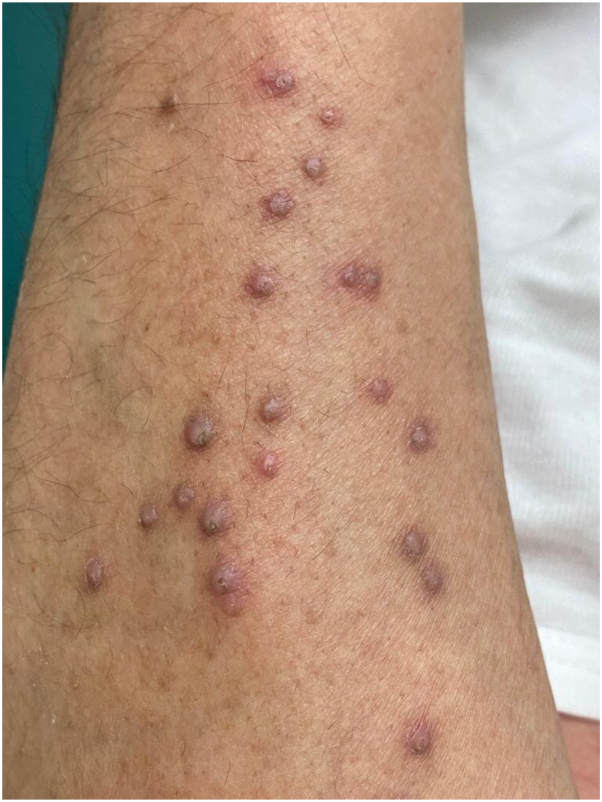
Multiple erythematous umbilicated papules with adherent central keratotic plugs over the extensor surface of the forearm.

A 45-year-old man with a 7-year history of generalized pustular psoriasis not controlled with various treatment regimens, including cyclosporine, methotrexate, and adalimumab. Six years following his initial presentation, the patient’s treatment was changed to risankizumab, an IL-23 inhibitor. He initially received a dose of 150 mg by subcutaneous injection. During a follow-up appointment 4 weeks later, the patient reported a remarkable improvement in his psoriasis, nearly achieving complete clearance. However, he subsequently noticed new asymptomatic lesions appearing on both forearms approximately 2 weeks after receiving the first dose of risankizumab. On close examination, these lesions were characterized by erythematous, umbilicated papules, each measuring less than 5 mm in diameter, with central adherent keratotic plugs. They were symmetrically distributed over the extensor surfaces of both forearms (Fig 1).

**Fig 2:**
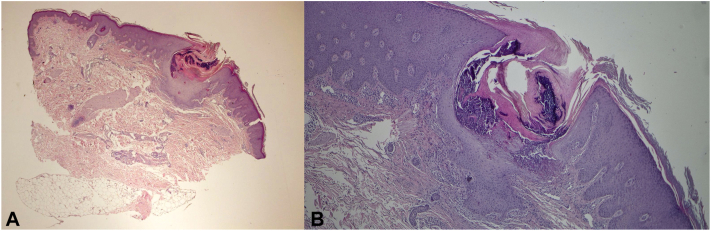
Hematoxylin-eosin staining show corneal pustule with psoriasiform hyperplasia, spongiosis of the epidermis and maintained granular layer (**A**), close-up view of vertically oriented elastic fibers penetrating the epidermis (**B**).

**Fig 3:**
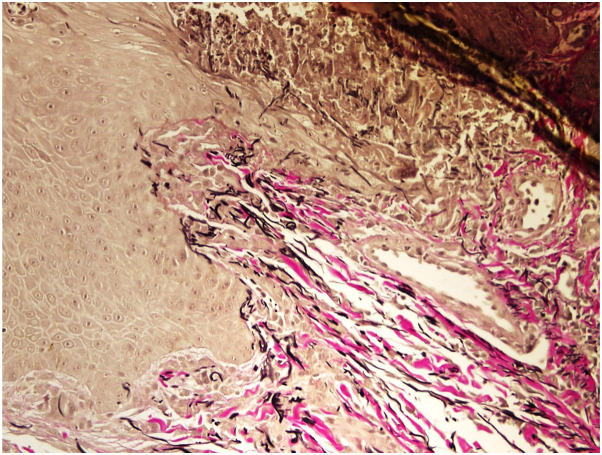
Elastic Verhoeff Van Gieson special stain highlighting the presence of elastic fibers within the epidermis.

Routine laboratory tests revealed normal renal function, but there were multiple instances of elevated hemoglobin A1C levels dating back to 2018, with the highest recorded being 9.3%. The patient had not previously been diagnosed with diabetes mellitus and had never received any related treatment. A 5-mm skin punch biopsy was performed under local anesthesia, and the specimen was sent for histological staining and examination. The histopathological analysis revealed the presence of corneal pustules with psoriasiform hyperplasia, spongiosis of the epidermis, and a preserved granular layer. In the center of the lesion, vertically oriented elastic fibers were observed penetrating the epidermis, along with degenerated neutrophils containing nuclear debris. In the superficial dermis, there was a perivascular lymphohistiocytic infiltrate with scattered neutrophils and eosinophils (Fig 2). Special stains using Elastic Verhoeff Van Gieson confirmed the presence of elastic fibers in the epidermis (Fig 3). The diagnosis was consistent with APD, specifically elastosis perforans serpiginosa. Because of the effective control of psoriasis, risankizumab was not discontinued. Four months later, all lesions spontaneously resolved without any residual signs. The patient has been monitored for 1 year, and no recurrence of similar lesions has been observed.

## Discussion

Perforating dermatoses encompass a category of skin disorders characterized by the penetration of dermal connective tissue through the epidermis.^1^ APD typically emerges in adulthood and may exhibit similarities to classic perforating disorders such as reactive perforating collagenosis, elastosis perforans serpiginosa, perforating folliculitis, or Kyrle’s disease.^1^^,^^2^

Clinical features of APD exhibit a lack of uniformity, with the most frequently reported presentations in the literature being hyperkeratotic papules and nodules, which tend to develop most frequently on the extensor surfaces of the extremities and trunk.^2^ APD lesions are typically associated with pruritus, although pain has been reported in a limited number of cases.^2^ Histopathological features are variable and may resemble any of the perforating dermatoses involving the transepidermal elimination of various dermal components, including collagen and elastin.^3^

The precise mechanism underlying the development of APD remains incompletely understood. However, it has been linked to various disease entities, with trauma resulting from pruritus and scratching believed to play a significant role.^1^ Diabetes and chronic renal failure are commonly associated with APD^4^ but it has also been observed in connection with a wide range of other conditions, including but not limited to hepatic and endocrinologic diseases, AIDS, and lymphoma.^5^, ^6^, ^7^ Furthermore, several medications have been reported as potential associations with APD, with the majority being newer biological agents. These include gefitinib, sorafenib, erlotinib, infliximab, etanercept, bevacizumab, natalizumab, and nilotinib.^8^

Rizakizumab is a humanized IgG1 monoclonal antibody that targets the p19 subunit of IL-23 and represents one of the recently FDA-approved biologics for treating psoriasis. The most frequently reported adverse events associated with the use of risankizumab include nasopharyngitis, upper respiratory tract infections, headache, gastroenteritis, diarrhea, back pain, and arthralgia.^9^ Cutaneous side effects linked to risankizumab are rare and encompass injection-site reactions and nonmelanoma skin cancer.^10^ However, it is worth noting that APD has not been previously reported in association with any of the IL-23 inhibitors.

Despite our patient’s previous history of elevated hemoglobin A1C levels over the past 5 years, the temporal relationship between the initiation of risankizumab therapy and the development of these lesions suggests a potential association. In addition, the fact that these lesions were asymptomatic in our case and devoid of clinical evidence of itching excludes scratching as a contributing factor.

In conclusion, we have presented a case of APD associated with the anti-IL23 agent risankizumab. Although APD is typically linked to systemic diseases rather than medications, there has been an increasing trend in reporting biologics as potential causes of this dermatosis. Further investigations are warranted to gain a more precise understanding of the underlying mechanisms involved.

## Conflicts of interest

None disclosed.
